# Dr. W. H. Atkinson

**Published:** 1891-08

**Authors:** 


### Dr. W. H. Atkinson.

The following is part of a paper entitled "As we go marching on," by J. F. Siddall, Oberlin, Ohio, read at the 32d Annual Meeting of the Northern Ohio Dental Association, of which Dr. Atkinson was a charter member.

"It remains for me to say a few words, in closing this paper, about the most honored member of our Association. I first met Wm. H. Atkinson in the Autumn of '57, or the Spring of '58, at a Dental Convention in Cleveland; then, again, I met him in a National Convention at Cincinnati, the following August, and I have remembered to this day more of Dr. Atkinson's appearance and things he said and did at that meeting than of any one else.

He seemed to be a stranger and would have taken but little part if his striking appearance and manner had not attracted attention, as always was the case with Dr. Atkinson. After those meetings he always remembered me, and seeing that I was anxious to learn, time and again the utmost cordiality, never failing to ask me "to come and look on."

Never in my life was I more thankful than for those opportunities. One incident I will relate that occurred years after, at a meeting of the State Society: A very delicate young woman was brought into our meeting with a large tumor in her mouth. She was seated in the center of a large circle of dentists who gathered about her.

Dr. Atkinson very carefully and rapidly made an examination of the case, and before he began his report to us dentists, he placed his hand gently on the head of the poor girl, who was a terrible looking object, and in a low tone of voice that I was close enough to hear, in a few simple words explained first to her the case, and assured her that Dr. Hamilton or any other good surgeon could with no difficulty whatever entirely remove the tumor, and that there was nothing to fear. The poor deformed sufferer who for a long time had been too shocking a sight to show her face even to her friends now animated with a new born hope and a beseeching look, together with the noble figure of Dr. Atkinson as he stood by her side, addressing the Society, with his hand still lovingly resting on her head like a tender father's, made a picture in my mind never to be forgotton.

The first thing I thought of when I heard of Dr. Atkinson's death was that scene, and the words of our Saviour, "Inasmuch as ye did it unto one of the least of these, ye did it unto me." How I wish our friend Dr. Barnes could have been there with his "Kodak," and could have given us that picture. It would have made a model indeed for an artist. This was but one, no doubt, of a great many incidents in the life of the good man where his skill or his counsel was so faithfully and lovingly bestowed as to be to the world a good representation of the Great Physician in his matchless power that never was witheld where there was human woe or want to relieve.

Dr. Atkinson also knew the blessedness of giving. How often have we heard him say, "Freely ye have received, freely give." With him it was give, give, give; his time, his thought, by day and by night, and with it went always his love, but last and least of all in his estimation often went his bottom dollar. This he gave so easily and so often that it was no trick at all.

The real value of such a life as that of Dr. Wm. H. Atkinson's to this association can not be estimated, to say nothing of what his life has been worth to other societies and to the profession at large. I once heard it remarked that Atkinson had impressed himself on the dental profession of the entire world as no other man that lived had done. This may be true or it may not.

We shall never be able to estimate the value of such a power as this man has been in our profession. We shall look in vain now for his remarks, so unlike any other man's, in the reports of Society proceedings in our Journals. His noble presence, an inspiration wherever seen, we shall behold no more. That manly form has gone, but who for a moment doubts that his noble "soul goes marching on" and shall not we who knew him, or knew of him, or have in any way been influenced by his teaching, and his life, all be the stronger for Atkinson's having lived, and for his still living; and shall not we, here and hereafter, with a firmer step and a brighter hope take courage "as we go marching on."

